# A new Early Cretaceous relative of Gnetales: *Siphonospermum simplex *gen. et sp. nov. from the Yixian Formation of Northeast China

**DOI:** 10.1186/1471-2148-10-183

**Published:** 2010-06-17

**Authors:** Catarina Rydin, Else Marie Friis

**Affiliations:** 1 University of Zürich, Institute of Systematic Botany, Zollikerstrasse 107, CH-8008 Zürich, Switzerland; 2 Swedish Museum of Natural History, Department of Palaeobotany, Box 50007, SE-104 05 Stockholm, Sweden

## Abstract

**Background:**

Knowledge on fossil and evolutionary history of the Gnetales has expanded rapidly; *Ephedra *and ephedroids as well as the *Gnetum-Welwitschia *clade are now well documented in the Early Cretaceous. However, hypotheses on evolutionary relationships among living and fossil species are hampered by restricted knowledge of morphological variation in living groups and recent studies indicate that gnetalean diversity and character evolution may be more complex than previously assumed and involve additional extinct groups (Bennettitales, Erdtmanithecales and unassigned fossil taxa).

**Results:**

Here we describe a new fossil related to Gnetales, *Siphonospermum simplex *from the Early Cretaceous Yixian Formation, an impression/compression of a reproductive shoot. The slender main axis bears one pair of opposite and linear leaves with primary parallel venation. The reproductive units are ovoid, without supporting bracts and borne on one median and two lateral branches. The most conspicuous feature of the fossil is the long, thread-like micropylar tube formed by the integument. Each ovule is surrounded by two different layers representing one or two seed envelopes; an inner sclerenchymatous layer and an outer probably parenchymatous layer.

**Conclusions:**

The vegetative and reproductive features of *Siphonospermum simplex *exclude a relationship to any other group than the Gnetales. A combination of opposite phyllotaxis, linear leaves and ovules surrounded by seed envelope(s) and with a long exposed micropylar tube are known only for extant and extinct Gnetales. *Siphonospermum simplex *constitutes a new lineage within the Gnetales. Its morphology cannot be directly linked to any previously known plant, but the organization of the reproductive units indicates that it belongs to the *Gnetum-Welwitschia *clade. Based on the absence of cone bracts and the inferred histology of the seed envelope(s) it could be related to *Gnetum*, however, there are also affinities with the ephedran lineage, some of which are likely plesiomorphic features, others perhaps not. Phylogeny and character evolution in the Bennettitales, Erdtmanithecales and Gnetales are currently only partly understood and under debate; the exact systematic position of *Siphonospermum simplex*, i.e., its position within the Gnetales, cannot be resolved with certainty.

## Background

The Gnetales comprise about 65-75 species in three genera (*Ephedra *L., *Gnetum *L., *Welwitschia *Hook. f.) [1]. It has long been assumed that the extant species are relictual remnants of a former greater diversity [[2], and others], and the rapidly expanding documentation of gnetalean fossils from the Early Cretaceous supports this idea. The Gnetales are reported from the Early Cretaceous of many parts of the world [3-16]. There are, in addition, fossils with a gnetalean affinity that are difficult to assign to any of the extant lineages [e.g., [17-20]] and a large diversity of pre-Cretaceous fossils, which have been discussed as potentially related to the Gnetales, but for which a precise systematic affinity remains to be established [e.g., [21,22]].

The members of the extant clade are characterized by several unique features [23-26], for example, decussate phyllotaxis, compound reproductive structures, polyplicate pollen and a single terminal unitegmic ovule with a resistant micropylar tube formed by the integument. The ovule is surrounded by structure(s) of bract origin (i.e., seed envelope[s]) and the micopylar tube reaches beyond the seed envelope and serves as the pollen receiving area [e.g., [26]]. *Ephedra *has retained many ancestral features, but can be defined by specialized pollen characters [27] and apical papillae on the seed envelope [28,29]. The sister group of *Ephedra*, the *Gnetum-Welwitschia *clade, is defined e.g., by the presence of an embryo feeder, higher order venation and many anatomical, histological and developmental details [23,30]. Assigning a fossil to either of the two major groups of the Gnetales is thus often straight forward due to the presence of these clear synapomorphies, but resolving higher level relationships among living and fossil taxa has proven difficult, partly due to restricted knowledge of phylogeny and morphological variation among living species. Further, it has lately been suggested that the Gnetales belong to a larger clade [the BEG-clade sensu Friis et al., see [31]], which also comprises the Bennettitales, the Erdtmanithecales and several unassigned fossil taxa [14,18,31]. The BEG hypothesis has been questioned [32]; interpretations of the integument in Bennettitales differ between authors, but nevertheless, evolutionary patterns in the Gnetales and potential stem lineages are obviously more complex than previously recognized.

In this study we add to the knowledge of gnetalean diversity in the Early Cretaceous. We describe and discuss an impression/compression fossil from the Liaoning province of north-eastern China collected in Early Cretaceous sediments of the Yixian Formation.

## Results

**Figure 1:**
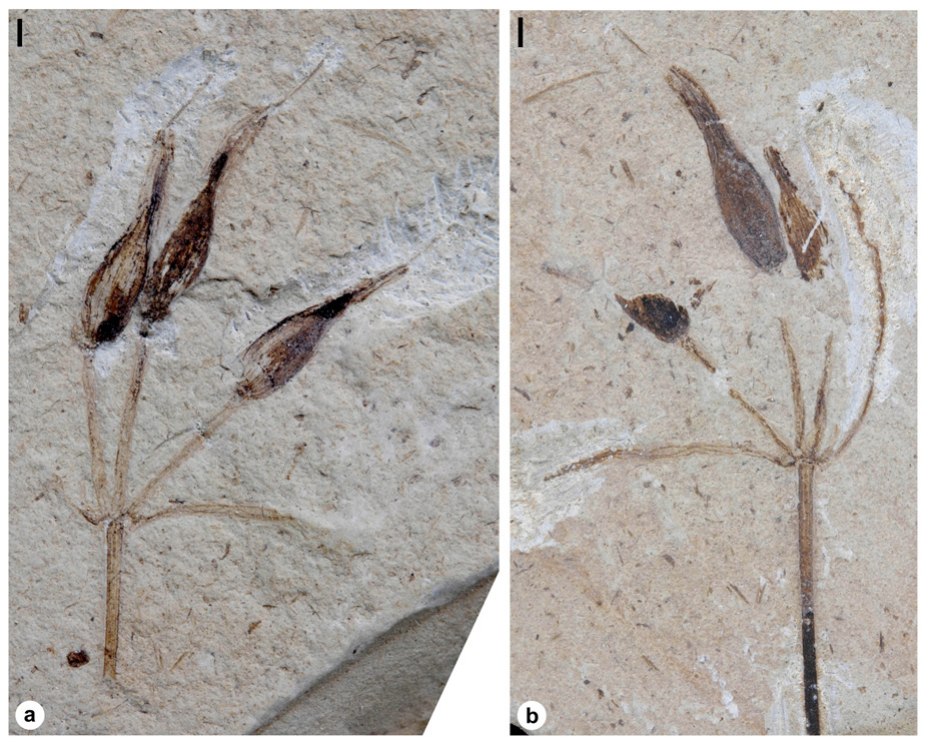
***Siphonospermum simplex *gen. et sp. nov**. Overview of the holotype (part and counterpart): (a) specimen 9880A; (b) specimen 9880B. Scale bars: 1 mm.

The type material of *Siphonospermum simplex *comprises two impression/compression fossils of a female plant, part and counterpart, with remnants of oxidized organic material (Figure 1). Few anatomical details are preserved, but lignified tissues can be observed. The total preserved length is about 25 mm. The branch bearing the cluster of ovules is about 0.5 mm thick and has one pair of opposite leaves. Leaves are linear, at least 10-13 mm long and 0.3-0.4 mm wide, and with remains of three primary veins. Three reproductive units are positioned terminally on one median and two lateral branches and are naked, without supporting bracts (Figure 1).

**Figure 2:**
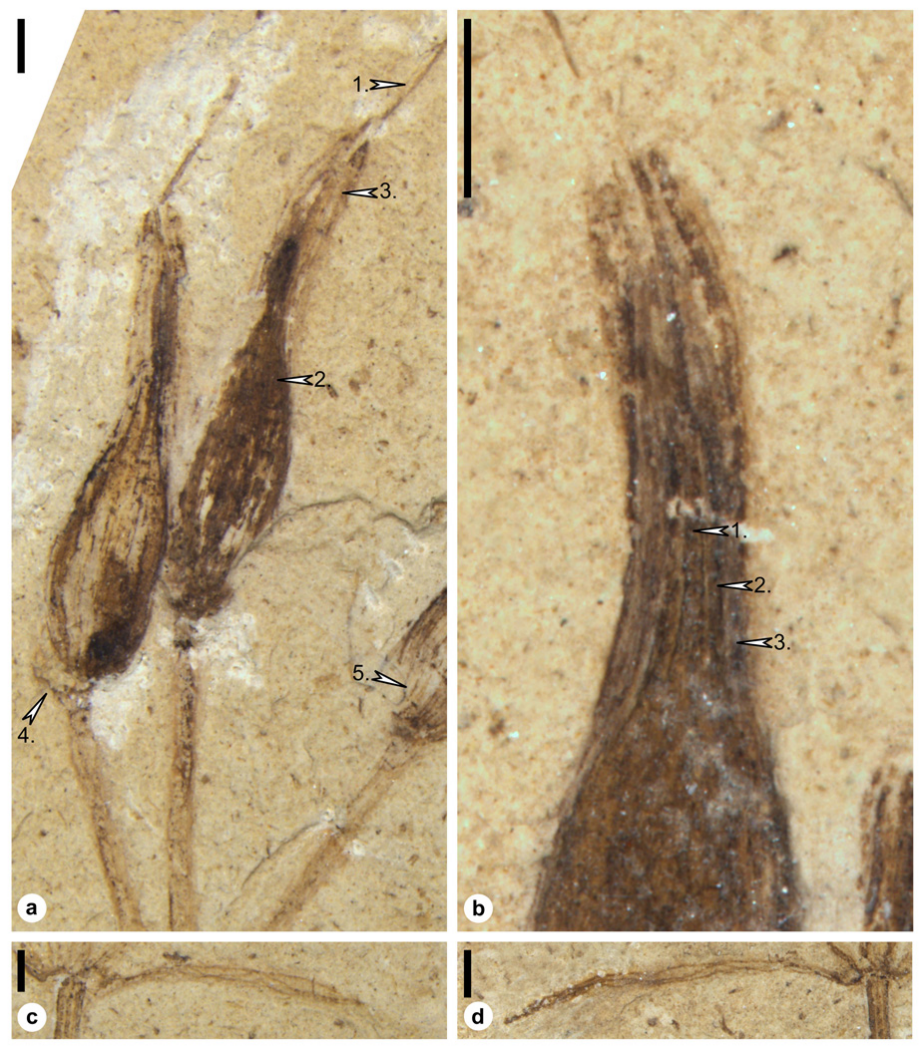
***Siphonospermum simplex *gen. et sp. nov**. Details of (a, c): specimen 9880A; (b, d): specimen 9880B. (a) Reproductive units of *Siphonospermum simplex*. (b) Close up of the reproductive unit seen in Figure 1b. (c-d) Node and linear leaf with parallel first order venation. Arrows 1: micropylar tube formed by the integument; 2: second (mostly sclerenchymatous) layer; 3: third (probably mostly parenchymatous) layer; 4: the ovule has been detached and slightly displaced; 5: supportive strands of the (inner) seed envelope. Scale bars: 1 mm.

**Figure 3:**
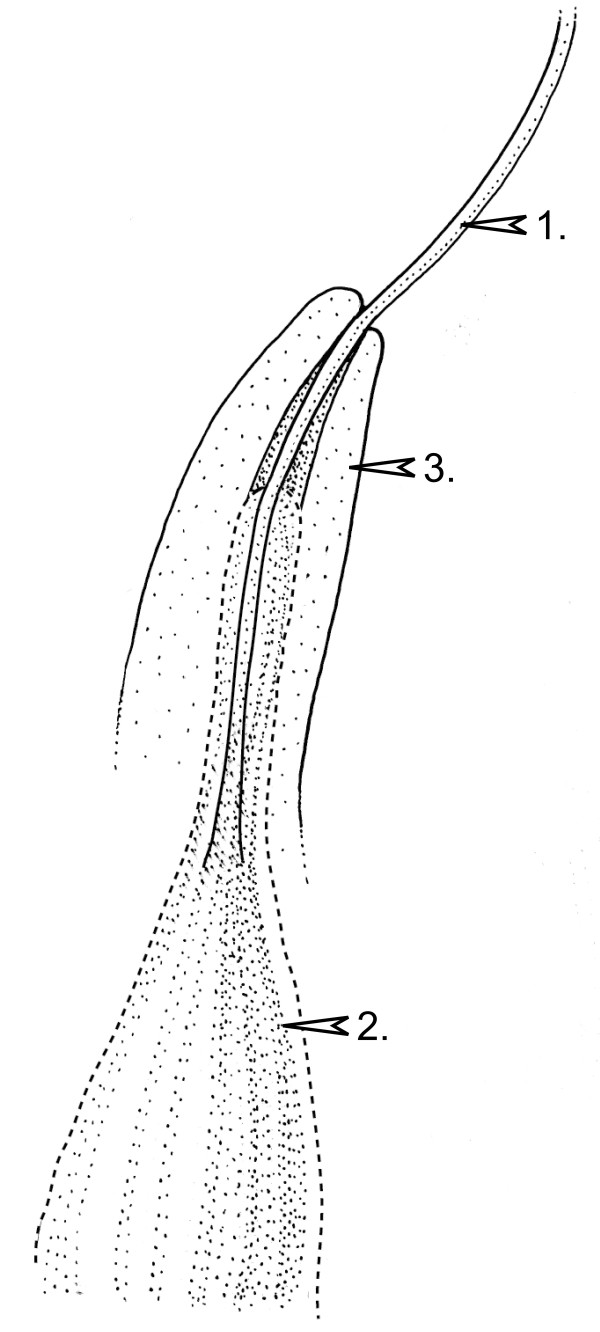
**Reconstruction of *Siphonospermum simplex***. Reproductive unit; reconstruction of a longitudinal section. Arrows 1: micropylar tube, 2: second (mostly sclerenchymatous) layer, 3: third (probably mostly parenchymatous) layer. Uninterrupted lines indicate morphological surfaces. The dashed line indicates the boundary between the second (mostly sclerenchymatous) layer and the third (probably mostly parenchymatous) layer. Drawing: Pollyanna von Knorring.

Reproductive units are about 8-9 mm long and 1.5-2 mm wide, narrowly ovoid with a rounded base and an extended acuminate apex (Figure 2a). Each ovule is surrounded by two distinct layers, which represent one or two seed envelopes (Figures 2b, Figure 3). The outermost layer is seen as an imprint in apical parts of the ovules. It is about 0.3-0.5 mm thick. The innermost layer appears massive with remnants of coaly matter. Numerous supportive strands form a pattern with dichotomies and anastomoses (Figure 2a). No papillae are observed on the seed envelope lining the micropylar tube.

There is a long thread-like micropylar tube that extends well above the surrounding seed envelope(s). The micropylar tube is straight, appears sturdy, and extends out of the seed envelope(s) at a slight angle. It is nearly equal in width throughout its length (Figure 2b); its diameter is about 0.2 mm proximally and about 0.1 mm distally. The total length of the micropyle is about 4.5-5.5 mm and the exposed part about 2.5-3.5 mm. The nucellus is not observed.

## Discussion

### Interpretation of the fossil and its systematic position

The vegetative and reproductive features of the new fossil exclude a relationship to any other group than the Gnetales. Ovules surrounded by one or two seed envelopes and with the integument extended into a long exposed micropylar tube are known only for extant and extinct Gnetales, for at least some members of the Bennettitales [14,18,31] and for a number of extinct plants thought to be closely related to Gnetales: i.e., the Erdtmanithecales [33] and a number of unassigned taxa based on dispersed seeds, such as *Buarcospermum*, *Lignierispermum*, *Lobospermum*, *Rugonella *[14]*Raunsgaardispermum *[18].

A close relationship with the Bennettitales can be ruled out based on vegetative as well as reproductive characters. The Bennettitales have ovules borne on long stalks, like *Siphonospermum simplex*, but the Bennettitales are otherwise distinct from *Siphonospermum *in having ovules borne in densely crowded heads and interspersed with fleshy interseminal scales. The Bennettitales are further distinguished by their typically larger and compound leaves. The Erdtmanithecales are known only from dispersed reproductive structures (seeds, male organs, pollen) [see summary e.g., in [13]]. The seeds of Erdtmanithecales are distinguished by their three-valved seed envelope, but further comparison with Erdtmanithecales is not possible.

Higher level relationship of *Siphonospermum simplex *(its systematic position within the Gnetales) is more difficult to establish due to the restricted amount of preserved information. Its morphology differs substantially from that of other gnetalean fossils from the Yixian Formation, which have compound cones comprising cone bracts and seeds [7,11,12]. They are generally interpreted as close relatives of *Ephedra*. In contrast, the reproductive units of *Siphonospermum *consist only of single ovules (with surrounding envelope[s]); there are no supporting bracts.

The long and conspicuous micropylar tubes of *Siphonospermum *are well preserved and have not been bent or curled during fossilization. Their walls are prominent (Figure 2b) and one tube (Figure 2a, left ovule) is broken at the apical level of the seed envelope. In *Ephedra*, *Gnetum *and *Welwitschia*, the inner epidermis of the micropylar tube is strongly thickened, probably lignified [[34], and pers. obs.], and the same appears to hold for *Siphonospermum*. Conceivably, the thickening and support for the micropylar tube have been important in the Gnetales in order to withstand visits from pollinators. Nothing is known about the pollination biology of *Siphonospermum*, but in extant Gnetales, pollination drops secreted via the micropylar tube serve as reward for pollinators (mainly Dipterans and Hymenopterans) [35-38]. Abiotic pollination is probably also important, at least in *Ephedra *[38,39].

Vegetative characters of *Siphonospermum simplex *are similar to extant *Ephedra*, with striate, erect stems and linear leaves opposite at nodes and with joined bases. However, these characters are not unique to *Ephedra*, but are general (ancestral) characters of the Gnetales, at least partly present also in *Gnetum*, *Welwitschia *and other gnetalean fossils [e.g., [4,12]]. The venation of the leaves of *Siphonospermum *is difficult to assess with certainty due to poor preservation and slightly folded leaves. There are three primary veins and perhaps also second order venation. The latter feature is interesting because it is a derived feature within the Gnetales, which characterizes the *Gnetum-Welwitschia *clade. Extant *Ephedra *species have two primary veins in leaves and cone bracts and so have most Early Cretaceous ephedroids such as *Liaoxia *[12,40]. One ephedroid fossil, *Liaoxia robusta *[12], has three or four primary veins but second order venation is unknown in fossil ephedroids as well as in extant *Ephedra*.

*Siphonospermum *has several features in common with *Gnetum*. The "reticulate" venation of *Gnetum *leaves develops from successive dichotomies in 5-10 parallel veins located in the centre of the leaf [41] and basically, the venation pattern of *Siphonospermum *is not different from that seen in *Gnetum*. The reproductive units of *Siphonospermum *have a similar shape as those of *Gnetum *and are (like in *Gnetum*) completely exposed with no remnants of cone bracts. Weak impressions of a structure at the lower left of the reproductive unit with the broken micropylar tube (Figure 2a, arrow 4) are not remains of a bract but reflect the original position of the ovule. This reproductive unit appears to have been detached and slightly displaced.

In *Gnetum *the nucellus is surrounded by three structures, an integument that forms the extending micropylar tube, a sclerenchymatous inner seed envelope and a second seed envelope, composed of parenchymatous cells and sclereids [42,43]. In *Siphonospermum*, the integument is surrounded by a persistent coalified tissue and its preservation mode indicates that it is sclerenchymatous. In the acuminate apex region, there are weak impressions of an additional tissue. There are no remains of sclerenchymatous tissue in this zone; it has obviously only left an imprint without cellular remains, which suggests soft tissue.

Further, the numerous supportive strands in the sclerenchymatous zone form a pattern with dichotomies and anastomoses, which is very similar to that formed by vascular bundles in the "endotesta" of *Gnetum *[44]. In the Gnetales, vascular bundles are absent in the integument but present in the seed envelopes [24], which supports our interpretation that the supportive strands of *Siphonospermum *are originally derived from the seed envelope.

### Conflicting information and uncertain evolutionary relationships

An evolutionary origin of the spikes of *Gnetum*, with sessile reproductive units arranged in whorls, from the pedunculate and solitary units of *Siphonospermum*, could be hypothesized by introducing hypothetical steps. However, there are several noteworthy differences between *Siphonospermum *and *Gnetum*, which make evolutionary relationships uncertain. First, the linear leaves and arrangements of the reproductive units are different from those of *Gnetum *(but likely plesiomorphic features in the Gnetales). Second, in *Gnetum *the micropylar tube is closed by radially expanding cells of the integument [45], whereas the micropylar tubes of *Ephedra *and *Welwitschia *are hollow [28,42] (and pers. obs.). In *Siphonospermum*, the micropylar tube is isodiametric and there are no indications of a closure tissue. Third, it is not possible to say if the outermost tissue of the reproductive units of *Siphonospermum *represents an additional seed envelope like in *Gnetum *[24], or an outer parenchymatous zone of a single seed envelope like in most species of *Ephedra *[34].

Thus, several seemingly "ephedroid" features of *Siphonospermum *may instead be plesiomorphic in the Gnetales, and retained in *Siphonospermum*. The general structure of the reproductive units indicates a sister relationship with *Gnetum*. If so, the closure tissue in the micropylar tube is a unique feature for *Gnetum *[and at least some species of Bennettitales [45]], not shared by *Siphonospermum *(or is missing in *Siphonospermum *because of an early stage of development). Alternatively, *Siphonospermum *could be sister to the *Gnetum-Welwitschia *clade; further studies of the diversity in the Gnetales and related taxa, and a better understanding of character evolution, are needed to elucidate higher level relationships among living and fossil species and the exact phylogenetic position of *Siphonospermum simplex*.

## Conclusions

The new Early Cretaceous impression/compression fossil described here, *Siphonospermum simplex*, possesses several key characters that suggest a close relationship with Gnetales and more specifically with the *Gnetum-Welwitschia *clade. Ovules borne without subtending cone bracts but with a possible second seed envelope indicate a relationship with *Gnetum*. However, there are also differences between *Gnetum *and *Siphonospermum*, most importantly the apparent absence of closure tissue in the micropylar tube.

Character evolution in the Gnetales and related extinct taxa is not fully understood. Often, a mixture of characters previously thought to be diagnostic for either the *Ephedra *lineage, the *Gnetum-Welwitschia *clade, or the Bennettitales, are seen in recently described charcoalified seeds [14,18] and the same is true for the new fossil presented here. *Siphonospermum simplex *clearly constitutes a new evolutionary lineage within the Gnetales, perhaps sister to *Gnetum *or to the *Gnetum-Welwitschia *clade, but its precise systematic position can currently not be resolved with certainty.

## Systematics

### Spermatophyta

Order: Gnetales

*Siphonospermum *gen. nov.

*Generic diagnosis*: Erect stem with terminal units of reproductive structures. Leaves linear, opposite at node, with primary parallel venation. Reproductive units without supporting bracts and terminal on median and lateral branches, narrowly ovoid with rounded base, extended acuminate apex, each consisting of one ovule, orthotropous and unitegmic. Ovules surrounded by an inner sclerenchymatous and an outer parenchymatous layer. Integument extended apically into a long thread-like micropylar tube.

*Etymology*: From the long thread-like micropyle (siphon) and seed (spermum).

*Type species: Siphonospermum simplex *sp. nov.

*Specific diagnosis*: As for the genus.

*Etymology*: From the simple architecture of the plant.

*Holotype*: 9880A (Figure 1a), 9880B (Figure 1b), housed in the palaeobotanical collections of the Institute of Botany, CAS, Beijing.

*Stratigraphic position and age*: Yixian Formation, Lower Cretaceous, (Early Aptian-early Late Aptian).

*Description and comments*: See results.

## Methods

The fossil material described here was collected from the Yixian Formation by a local collector and occurs as part and counterpart on a slab of finely laminated light grey to yellowish siltstone. The fossil is preserved as compressions/impressions with only little organic material remaining. No detailed locality information was attached to the specimen, but examination of the lithology suggests that the fossil was collected from the Jianshangou locality (Beipiao, Chaoyang City, west Liaoning) from the lower part of the Yixian Formation (Wang Xiaolin, personal communication 2006). In the present work we leave the exact provenance open and refer broadly to the Yixian Formation of Northeast China.

The Yixian Formation is widely distributed in the western part of the Liaoning Province, but outcrops also in northern Hebei and Inner Mongolia. The age is established by radiometric dating to about 125 - 120 Ma [see summary in [46]], corresponding to the Early Aptian-earliest Late Aptian [47]. The exceptionally rich fauna and flora are important constituents of the Jehol Biota. The fauna is particularly well preserved and includes a diversity of invertebrates, osteichthyan fish, amphibians, mammals and reptiles including feathered dinosaurs and early birds [48]. The flora is also diverse [7,49], but generally the preservation of the plant fossils is not as good as for the fossil animals. Fossil plants related to Gnetales are particularly diverse [7,11,12,40] (and the new fossil described here). Most gnetalean fossils are ephedroid, but none of them possesses defining characters of extant *Ephedra *and most of them have been assigned to various species of the extinct genus *Liaoxia*.

To comply with requirements of the ICBN, we have deposited paper copies of this article at the University of Zürich (library of the Institute of Systematic Botany); Stockholm University (library of the Department of Botany); Peking University Library, Beijing; Chinese Academy of Sciences, Beijing (library of the Institute of Botany); and the Swedish Museum of Natural History (library of the Department of Palaeobotany).

## Authors' contributions

CR and EMF conducted data analyses and evolutionary interpretations. CR wrote the manuscript and EMF provided extensive contributions to the text. All authors read and approved the final manuscript.
